# Inducing Toxicity in the Neurological System by Environmental Pollutants

**DOI:** 10.3390/toxics12070480

**Published:** 2024-06-30

**Authors:** Irene Camacho, Isabel Fragoeiro, Daniel Neto

**Affiliations:** 1 Faculty of Life Sciences, Madeira University, Campus Universitário da Penteada, 9020-105 Funchal, Portugal; 2 Escola Superior de Saúde, Madeira University, Campus Universitário da Penteada, 9020-105 Funchal, Portugal; isabel.fragoeiro@staff.uma.pt; 3 Chronic Diseases Research Center CEDOC, NOVA Medical School, Faculdade de Ciências Médicas, Universidade NOVA de Lisboa, Campo Mártires da Pátria 130, 1099-085 Lisbon, Portugal; danielcarvalhoneto@gmail.com

There is a growing body of evidence that exposure to multiple air pollutants may cause dysfunction of the central nervous system (CNS). According to the Lancet Commission on pollution and health, in recent years, air pollution has become the largest environmental contributor of illness and early death worldwide [1].

Neurotoxic pollutants can be toxic to fetuses, infants, and children due to the vulnerability of the developing brain at the early stages, also causing damage in the elderly population. Research shows cause–effect associations between particulate matter pollution and common non-contagious diseases, namely impairment of cognitive function, attention deficit disorder, dyslexia, autism and behavioural disorders in childhood. On the other hand, neurodegenerative diseases, which include dementia symptoms in adults, have been reported. Several epidemiologic studies have shown that exposure to traffic-related air pollutants poses a risk for Alzheimer’s disease and several neurodevelopmental disorders, such as attention deficit hyperreactivity disorder, intellectual disabilities and schizophrenia [1,2]. There is evidence that highly polluted and industrialized regions can also be deleterious for neurological development, particularly in child populations, and can cause different sorts of neurobehavioral deficits (Contribution 2).

Autism spectrum disorder and Alzheimer’s disease constitute common neurological diseases that may indicate the interactions between environmental influences and genetic susceptibilities. Emerging human and animal studies have helped to show the underlying molecular mechanisms of susceptibility and disease. Inflammation and oxidative stress mechanisms are regarded as the major physio-pathological process underlying brain damage due to air pollution exposure. Air pollutants can act in the CNS by cellular, molecular and inflammatory pathways. In fact, an array of several molecules that function as pro-inflammatory mediators and reactive oxygen species (ROS) can be produced in response to exposure to different air pollutants [3].

The purpose of the present Special Issue is to provide further insights into environmental pollutants that are affecting human health, particularly at a neuropathological level. The Special Issue primarily addresses exposomes, pollution sources, susceptibility profiles, and the mechanisms of the toxic actions of pollutants. A total of five high-quality articles have been accepted and are listed below. 

Lopez et al. present a paper entitled “Lung-Based, Exosome Inhibition Mediates Systemic Impacts Following Particulate Matter Exposure” (Contribution 4). In this study, the mechanistic role of lung-derived exosomes after PM exposure and the consequents impacts on the neurovascular system using a mice model were assessed. In order to do so, the authors used an exosome inhibitor (GW4869) to inhibit exosome formation in the lungs of mice via oropharyngeal aspiration. Then, we assessed the downstream behavioural, cellular, and molecular biomarkers in the lung, serum, and brain tissue of the exposed mice, who had previously inhaled particulate matter (PM) from a legacy mine-site. The results suggest the role of exosomes in driving cerebrovascular impairment following PM exposure, which is mitigated with GW4869 lung-based administration.

Calderón-Garcidueñas et al. present a study entitled “Environmentally Toxic Solid Nanoparticles in Noradrenergic and Dopaminergic Nuclei and Cerebellum of Metropolitan Mexico City Children and Young Adults with Neural Quadruple Misfolded Protein Pathologies and High Exposures to Nano Particulate Matter” (Contribution 3). In this study, environmental solid nanoparticles (NPs) reaching noradrenergic and dopaminergic nuclei and the cerebellum were investigated, in addition to their associated ultrastructural alterations in post-mortem samples derived from the metropolitan Mexico City. NPs were detected in the locus coeruleus, substantia nigrae and cerebellum. The results showed an NP profile with Fe, Ti, W, Hg and Zn in such tissues and in several cellular organelles, along with early and progressive neurovascular damage and cerebellar endothelial erythrophagocytosis. Based on these findings, the authors stress that NP exposures in early life pose a high risk to brain development and lethal neurologic outcomes. Therefore, exposed children and young adults require early neuroprotection and NP/specific metal sources ought to be clearly identified, regulated, and assessed.

Abdulaziz et al. present the work entitled “The Influence of Photodynamic Antimicrobial Chemotherapy on the Microbiome, Neuroendocrine and Immune System of Crustacean Post Larvae” (Contribution 1). They evaluated the action of the photosensitizer curcumin on the microbiome and neuroendocrine and immune systems of *Penaeus monodon*, a marine crustacean also known as the giant tiger prawn. Photodynamic therapy using curcumin as a photosensitizer has been tested to kill water-associated pathogens that cause diseases to aquatic animals and seafood spoilage. The authors recommend photodynamic antimicrobial chemotherapy with curcumin as a photosensitizer to disinfect water-associated pathogens in the larval rearing system of P. monodon. Nevertheless, it was shown that curcumin and photoexcited curcumin are able to alter the expression levels of two hormones: the moult-inhibiting and the crustacean hyperglycaemic hormone, which may affect the growth and survival rate of these animals. Hence, photodynamic therapy should not be used in aquaculture systems when animals undergo moulting or are subjected to any type of environmental stress. In addition, the duration of photoexcitation should be kept to a minimum to avoid the build-up of ROS beyond the tolerance limit of the organisms being cultivated.

Finally, two papers close the set of articles of this Special Issue. Armas and D’Angiulli provide a complete and concise literature review on the effects of air pollution on the life-span development of the CNS and its functions. The work also discusses the occurrence of particulate concentrations in megacities and the possible mechanisms underlying neuroinflammation following significant and severe exposures to such substances. The authors concluded by associating the developmental processes in children with possible associated neuropathological consequences in adults and the elderly. In turn, Olasehinde and Olaniran provide another paper entitled: “Neurotoxicity of Polycyclic Aromatic Hydrocarbons: A Systematic Mapping and Review of Neuropathological Mechanisms” (Contribution 5), which details a systematic review that assessed the research trends associated with Polycyclic Aromatic Hydrocarbon (PAH)-induced neurotoxicity, providing awareness about their mechanisms of action and new therapeutic strategies. This is another relevant study because PAHs represent a class of environmental pollutants that find their way into humans via food and dietary sources, soil, drinking water, etc., and they are capable of causing neurological deficits. The work provides an interesting overview of the effect of PAHs on the CNS using a bibliometric approach and highlights the neuropathological mechanisms of PAHs.
